# From Iodine Maps to Brain Window Images: Quantitative Visibility Thresholds of Iodine Staining Using Photon-Counting CT—A Retrospective Observational Study

**DOI:** 10.3390/diagnostics16182915

**Published:** 2026-09-09

**Authors:** Marie-Christine Pali, Stephanie Mangesius, Constantin Emanuel Eisenschink, Philipp Deisl, Lukas Neumann, Michael Knoflach, Raimund Pechlaner, Bernhard Glodny, Lukas Lenhart, Elke Ruth Gizewski, Astrid Ellen Grams

**Affiliations:** 1 Department of Radiology, Medical University of Innsbruck, Anichstrasse 35, 6020 Innsbruck, Austria; marie-christine.pali@i-med.ac.at (M.-C.P.); constantin.eisenschink@i-med.ac.at (C.E.E.); philipp.deisl@i-med.ac.at (P.D.); bernhard.glodny@i-med.ac.at (B.G.); lukas.lenhart@i-med.ac.at (L.L.); elke.gizewski@i-med.ac.at (E.R.G.); astrid.grams@i-med.ac.at (A.E.G.); 2 Neuroimaging Research Core Facility, Medical University of Innsbruck, Anichstrasse 35, 6020 Innsbruck, Austria; 3 Department of Basic Sciences in Engineering Sciences, University of Innsbruck, 6020 Innsbruck, Austria; lukas.neumann@uibk.ac.at; 4 Department of Neurology, Medical University of Innsbruck, 6020 Innsbruck, Austria; michael.knoflach@i-med.ac.at (M.K.);

**Keywords:** photon-counting computed tomography, acute ischemic stroke, mechanical thrombectomy, iodine maps, virtual non-contrast imaging, brain imaging, quantitative imaging, contrast staining

## Abstract

**Objectives:** We aimed to determine quantitative photon-counting CT (PCCT)-derived iodine map (IM) attenuation associated with visual detectability of parenchymal hyperattenuation on conventional brain window (BW) images after mechanical thrombectomy (MT) and to assess corresponding relative BW attenuation changes. **Methods:** In this retrospective single-center study, 12 patients with anterior circulation large vessel occlusion underwent MT followed by post-interventional PCCT. BW, IM, and virtual non-contrast reconstructions were generated. Follow-up non-contrast CT served as reference for final infarction extent. Of 54 ASPECTS regions with final infarction, 40 showed IM hyperattenuation and were included in the detectability analysis. Two blinded readers assessed visual detectability. IM attenuation and relative BW attenuation increase (%ΔHU) were evaluated using ROC analysis with clustered bootstrap resampling. Thresholds were determined using the Youden index. **Results:** Across all 54 regions with final infarction, median IM attenuation was significantly higher in infarcted than contralateral regions (6.39 vs. 2.18 HU, *p* < 0.001), while absolute BW attenuation did not differ significantly. Among the 40 regions with IM hyperattenuation, 15 were visually detectable on BW images. ROC analysis showed excellent performance for IM (AUC = 0.997) and %ΔHU (AUC = 0.984). The optimal IM threshold in the original cohort was 8.33 HU (sensitivity 100%, specificity 96.0%), and the optimal %ΔHU threshold was 3.06% (sensitivity 100%, specificity 92.0%). Bootstrap-derived median thresholds were 11.69 HU (95% interval 8.33–12.67) for IM and 3.06% (3.06–20.16%) for %ΔHU. Inter-reader agreement was excellent (κ = 0.81–0.86). **Conclusions:** PCCT-derived iodine quantification enables objective assessment of visual detectability of post-interventional parenchymal hyperattenuation. In this cohort, an internally derived IM cutoff of 8.33 HU was associated with BW visibility. These preliminary findings require validation in larger, independent multicenter cohorts.

## 1. Introduction

Acute ischemic stroke caused by large vessel occlusion (LVO) of the anterior circulation is a leading cause of death and long-term disability. Mechanical thrombectomy (MT) in appropriately selected patients improves functional outcome and is established as standard-of-care therapy. Contemporary guidelines endorse MT, based on multiple randomized trials and subsequent evidence syntheses [1]. Despite successful reperfusion, early post-interventional imaging remains essential to identify hemorrhagic transformation, blood–brain barrier (BBB) disruption, and early ischemic injury. Conventional non-contrast computed tomography (NCCT) is widely used because of speed and accessibility. However, interpretation of post-MT CT can be challenging. Iodinated contrast administered during angiography may extravasate into brain parenchyma through a disrupted BBB, resulting in hyperattenuation that may mimic or coexist with intracranial hemorrhage. Moreover, lower levels of iodine accumulation may produce only subtle attenuation changes that are difficult to perceive on conventional brain window (BW) images [2]. Dual-energy computed tomography (DECT) has substantially improved the characterization of these post-interventional parenchymal abnormalities. By exploiting energy-dependent differences in X-ray attenuation, DECT enables material decomposition and the generation of iodine maps (IMs) and virtual non-contrast (VNC) images. IMs directly demonstrate and quantify iodine accumulation, whereas VNC reconstructions allow iodine-related attenuation to be distinguished from hemorrhage, thereby improving differentiation between contrast staining and blood after MT [3,4]. These capabilities have established DECT as a particularly valuable technique in the immediate post-MT setting. Beyond qualitative material differentiation, previous DECT studies have demonstrated that the magnitude of iodine accumulation contains clinically relevant quantitative information. Increased iodine concentration within post-interventional parenchymal hyperattenuation has been associated with subsequent hemorrhagic transformation, and quantitative iodine thresholds have been proposed for risk stratification [5,6]. In addition, quantitative IM and VNC attenuation have been shown to predict subsequent infarct development in areas of BBB disruption [3]. Thus, iodine-related attenuation is increasingly recognized not merely as a means of identifying contrast staining, but as a quantitative imaging biomarker reflecting the degree of BBB disruption and tissue injury. Importantly, however, previous quantitative thresholds have primarily been developed to predict biological or clinical outcomes, such as subsequent hemorrhage or infarction. Considerably less attention has been paid to the relationship between quantitatively measured iodine accumulation and its visual manifestation on BW images. The attenuation displayed on a routine BW image represents the combined contribution of underlying tissue and retained iodine. Consequently, iodine may already be quantitatively detectable on spectral material maps while its contribution to overall attenuation remains insufficient to produce a visually recognizable hyperattenuation. Establishing this relationship is relevant because routine clinical interpretation still relies heavily on conventional BW reconstructions, even when spectral datasets are available. Photon-counting computed tomography (PCCT) represents the newer generation of spectral CT technology. In contrast to conventional energy-integrating detector CT, PCCT detectors directly detect individual X-ray photons and discriminate them according to energy. This provides improved spatial resolution and spectral separation and enables energy-resolved reconstruction from each acquisition. These properties are particularly advantageous for material decomposition and quantitative iodine imaging [7,8,9,10]. Early experimental and clinical studies have demonstrated the feasibility of PCCT-based VNC imaging and iodine decomposition for differentiating iodinated contrast from intracranial hemorrhage, including in the post-MT setting [7,8].

The improved spectral capabilities of PCCT provide an opportunity to investigate the relationship between quantitative iodine signal and conventional image perception more precisely. In clinical practice, regions with marked iodine accumulation on PCCT-derived IM may remain isodense or only subtly hyperattenuating on corresponding BW reconstructions. However, to the best of our knowledge, the quantitative transition at which PCCT-measured iodine accumulation becomes visually perceptible on conventional BW images has not been systematically established. Such a perceptual threshold would provide a quantitative link between spectral material decomposition and conventional grayscale image interpretation and could help standardize interpretation of post-interventional CT findings across readers and clinical environments. Therefore, the aims of this study were (1) to determine a PCCT-derived IM threshold in Hounsfield Units (HU) above which post-interventional parenchymal iodine staining becomes visually detectable on conventional BW images and (2) to determine the minimum relative increase in BW attenuation associated with visual detectability. We hypothesized that PCCT-derived iodine concentration would provide a robust quantitative predictor of visual conspicuity and that iodine-based thresholds would be more stable than attenuation-based thresholds derived from conventional BW images.

The study was designed, conducted, and reported in accordance with the STROBE (Strengthening the Reporting of Observational Studies in Epidemiology) Statement for cohort studies and the SAGER (Sex and Gender Equity in Research) guidelines.

## 2. Materials and Methods

### 2.1. Study Population and Imaging

This retrospective study included 12 consecutive patients recruited between October 2025 and April 2026. Inclusion criteria were acute ischemic stroke due to anterior circulation LVO, treatment with MT, and subsequent post-interventional PCCT imaging (NAEOTOM Alpha Peak, Siemens Healthineers, Forchheim, Germany). According to the institutional post-MT imaging protocol, all patients undergoing MT routinely receive PCCT as the immediate post-interventional CT examination. Thus, the use of PCCT was not based on patient characteristics, procedural factors, or clinical status. Exclusion criteria were posterior circulation LVO, absence of MT, and severe movement artifacts on PCCT imaging.

From the PCCT acquisition, BW series, IM series, and VNC series were generated using the manufacturer’s clinical reconstruction pipeline. Material decomposition and iodine quantification were performed in accordance with established multi-energy CT principles as outlined by the AAPM Task Group 291 report [11]. Additionally, follow-up (FU) NCCT imaging was acquired, approximately 24 h after the post-interventional PCCT. From this imaging study, the final infarction extent was determined using the Alberta Stroke Program Early CT Score (ASPECTS). No missing imaging data were present for the variables included in the analysis.

The study was conducted in accordance with institutional and ethical guidelines and was approved by the Ethics Committee of the Medical University of Innsbruck (approval number 1188/2023; date: 21 September 2023).

### 2.2. Visual Analysis

**Figure 1:**
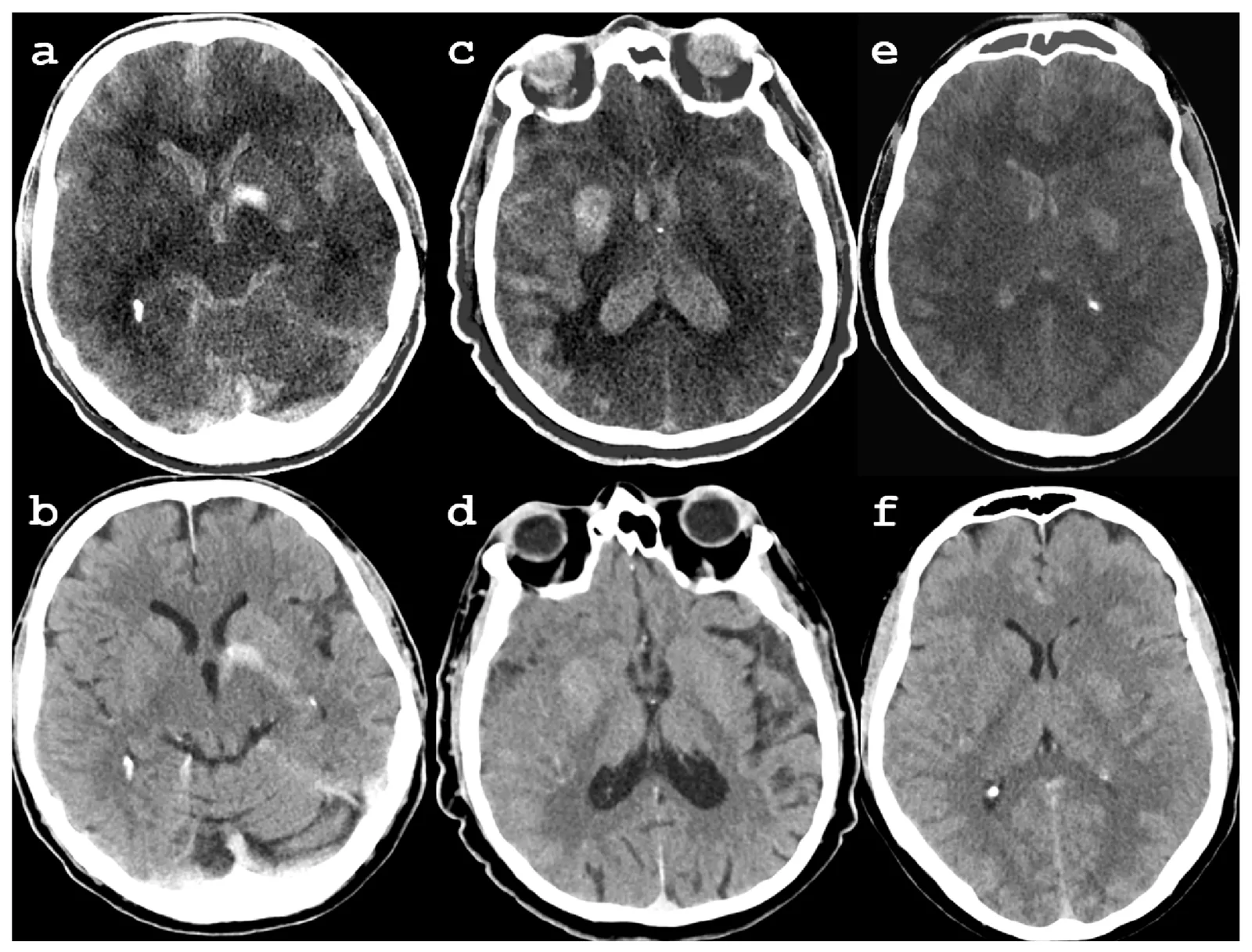
Examples of IM hyperattenuating areas that are clearly hyperattenuating on BW images (**a**,**b**), only mildly hyperattenuating on BW images (**c**,**d**), or not clearly hyperattenuating on BW images (**e**,**f**).

Visual detectability of parenchymal hyperattenuation on standard BW and IM images was independently assessed by two experienced readers (A.E.G., S.M.), who were blinded to the respective other reconstruction and to FU imaging (Figure 1). The detectability analysis was restricted to ASPECTS regions showing hyperattenuation on IM. For each IM-positive ASPECTS region, the readers assessed whether the corresponding parenchymal hyperattenuation was visually detectable on BW images and classified it as visually detectable or visually undetectable. The subsequent perceptual threshold analysis specifically assessed BW visibility among IM-positive regions rather than across all ASPECTS regions with final infarction. Post-interventional hemorrhage was assessed using VNC images. Regions demonstrating hyperattenuation on VNC images, corresponding to suspected post-interventional hemorrhage, were excluded from the present analysis. Discrepancies between the two readers were recorded and resolved by consensus for subsequent analyses.

### 2.3. Quantitative Assessment

Three circular Regions of Interest (ROIs) with an area of 10 mm^2^ were manually placed within each affected ASPECTS region. Corresponding contralateral reference ROIs of the same size were placed in the anatomically corresponding region of the opposite hemisphere at the same axial level. For each ASPECTS region, the values obtained from the three ROI measurements were averaged to obtain a single representative value for that region. The same procedure was applied to the corresponding contralateral measurements. Thus, individual ROI measurements were considered replicate measurements and were not treated as independent observations. Care was taken to avoid cerebrospinal fluid spaces, large vessels, calcifications, and image artifacts.

### 2.4. Statistical Analysis

Statistical analyses were performed using Python (version 3.13, Python Software Foundation, Beaverton, OR, USA), with SciPy and scikit-learn libraries, on an ASPECTS-region level. The three replicate ROI measurements within each ASPECTS region were averaged before statistical analysis, yielding one representative value per region. Thus, the hierarchical data structure consisted of ASPECTS regions nested within patients, while individual ROI measurements were not treated as independent observations. Because multiple ASPECTS regions originated from the same patient, clustering at the patient level was accounted for in diagnostic performance analyses using patient-level clustered bootstrap resampling.

Continuous variables were assessed for normality using the Shapiro–Wilk test. Normally distributed variables are reported as mean ± standard deviation, and non-normally distributed variables as median with interquartile range (IQR).

For each ASPECTS region, paired differences between infarct ROIs and contralateral control ROIs were calculated for IM values and BW attenuation values. Normality for paired comparisons was assessed using the distribution of the paired differences between infarct and contralateral ROIs. If paired differences were normally distributed, comparisons were performed using paired *t*-tests and are reported as mean paired difference with a 95% confidence interval (CI). If normality was not met, the Wilcoxon signed-rank test was used and results are reported as median paired difference.

For each infarct ROI, the relative attenuation increase on BW images compared to the contralateral ROI was calculated as:

$$\%\Delta HU=\frac{HU_{infarct}-HU_{contralateral}}{HU_{contralateral}}\times 100$$

This percentage difference served as the quantitative variable for determining the perceptual threshold for visual detectability of hyperattenuating infarct areas on standard BW images.

To identify quantitative thresholds associated with visual detectability, receiver operating characteristic (ROC) analyses were performed using IM values and relative BW attenuation increase (%ΔHU) as predictors, with consensus visual detectability (yes/no) as the binary reference standard. ROC analyses were restricted to ASPECTS regions showing hyperattenuation on IM.

Diagnostic performance was summarized using the area under the ROC curve (AUC) with 95% CI. The optimal cutoff value in the observed study cohort for each predictor was determined using the Youden index. Sensitivity, specificity, positive predictive value (PPV), and negative predictive value (NPV) were calculated for the identified cutoffs.

Because multiple ROIs originated from the same patient, patient-level clustered bootstrap resampling (2000 iterations) was used to account for within-patient clustering and to estimate 95% CIs for AUC values and cutoff-based performance metrics. Bootstrap CIs were calculated using the percentile method. The stability of the ROC-derived cutoff values was assessed based on the distribution of Youden-index-derived cutoffs across the bootstrap samples. These thresholds were considered internally derived candidate perceptual thresholds.

Inter-reader agreement for visual detectability on BW images was assessed using Cohen’s κ statistics and interpreted according to established guidelines (poor <0.20, fair 0.21–0.40, moderate 0.41–0.60, good 0.61–0.80, excellent >0.80).

All statistical tests were two-sided, and a *p*-value of less than 0.05 was considered statistically significant.

## 3. Results

A total of 12 patients were included in the analysis, comprising 5 male and 7 female patients. The mean age was 71.2 ± 12.9 years (median, 74 years; range, 47–87 years). The median time from symptom onset to MT was 233 min (mean, 284.4 ± 134.4 min; range, 155–577 min). The baseline National Institutes of Health Stroke Scale (NIHSS) score was 16.6 ± 4.8 (median, 18; range, 6–22). Intravenous thrombolysis was administered in eight patients (66.7%), whereas four patients (33.3%) did not receive intravenous thrombolysis.

Regarding stroke etiology, six patients (50.0%) had cryptogenic stroke, three (25.0%) had atrial fibrillation, two (16.7%) had ICA stenosis, and one patient (8.3%) had an aortic plaque. Occlusion sites included the M1 segment in seven patients, the carotid terminus in four patients, and the M2 segment in one patient. Two patients had an M1 occlusion associated with ICA stenosis. Initial ASPECTS was 9.1 ± 1.7 (median, 10; range, 4–10), decreasing to 5.7 ± 3.1 (median, 7; range, 0–9) at the final assessment.

All patients had an initial expanded TICI (eTICI) score of 0. Final angiographic reperfusion was graded as eTICI 3 in five patients (41.7%), eTICI 2c in one patient (8.3%), and eTICI 2b in six patients (50.0%). Stent retriever (SR) alone was used in four patients (33.3%), whereas aspiration and stent retriever (A&SR) were used in eight patients (66.7%). According to the Heidelberg Bleeding Classification, no intracranial hemorrhage was observed in nine patients (75.0%); HI1 was reported in one patient (8.3%), and class 3c bleeding in two patients (16.7%). Detailed patient, stroke and therapy characteristics of the 12 included patients are given in Supplementary Table S1.

A total of 54 ASPECTS regions with final infarction were identified in FU imaging. Hyperattenuating areas on IM series were identified in 40 (74.1%) of the 54 infarct regions. These 40 IM-positive regions constituted the primary analysis cohort for assessment of BW visual detectability. Among the 40 IM-positive regions, 15 (37.5%) were visually detectable as parenchymal hyperattenuation on BW images, whereas 25 (62.5%) were visually undetectable.

### 3.1. Inter-Reader Agreement

Inter-reader agreement for visual detectability of parenchymal hyperattenuation on IM series was excellent (κ = 0.81) and similarly high for the visibility assessment on BW series (κ = 0.86), indicating a robust reproducibility of visual classification between readers.

### 3.2. Quantitative Comparison of Infarct and Contralateral ROIs

Across all 54 infarct regions identified on FU imaging, IM values within infarct ROIs were not normally distributed (Shapiro–Wilk *p* = 0.0015) and thus reported as median (IQR). Median IM attenuation was 6.39 HU (IQR, 3.94–11.22) in infarct ROIs, compared with 2.18 HU (IQR, 0.66–3.56) in the contralateral ROIs. Paired differences were also not normally distributed (Shapiro–Wilk *p* = 0.0017). Therefore, the Wilcoxon signed-rank test was used, demonstrating a significant difference between the affected and contralateral regions, with a median paired difference of 4.01 HU (*p* < 0.001). The patient-level bootstrap 95% percentile interval for the median paired difference was 2.05–7.48 HU.

BW attenuation values were normally distributed in the individual infarct and contralateral measurements (Shapiro–Wilk *p* = 0.225 and *p* = 0.261, respectively). Mean BW attenuation was 39.20 ± 8.14 HU in infarct ROIs and 39.28 ± 3.18 HU in contralateral ROIs. The paired differences were not normally distributed (Shapiro–Wilk *p* = 0.0097), with no significant side-to-side difference on Wilcoxon signed-rank testing (median paired difference −0.32 HU, *p* = 0.565). The patient-level bootstrap 95% percentile interval for the median paired difference was −4.40 to 1.57 HU.

Within the cohort of 40 IM-positive regions, median IM attenuation was 7.58 HU (IQR, 5.63–12.97) in infarct ROIs and 2.18 HU (IQR, 0.76–3.39) in contralateral ROIs. The paired differences were approximately normally distributed (Shapiro–Wilk *p* = 0.081), and a paired *t*-test demonstrated a significant side-to-side difference, with a mean paired difference of 7.43 HU (95% CI, 5.29–9.56 HU; *p* < 0.001). The patient-level bootstrap 95% percentile interval for the mean paired difference was 4.76–11.19 HU.

For BW attenuation, mean values were 40.92 ± 8.41 HU in infarct ROIs and 39.10 ± 3.38 HU in contralateral ROIs. The paired differences were not normally distributed (Shapiro–Wilk *p* = 0.0065). Wilcoxon signed-rank testing showed no significant side-to-side difference, with a median paired difference of −0.02 HU (*p* = 0.372). The patient-level bootstrap 95% percentile interval for the median paired difference was −2.07 to 4.55 HU.

### 3.3. Quantitative Attenuation Stratified by BW Visual Detectability

**Figure 2:**
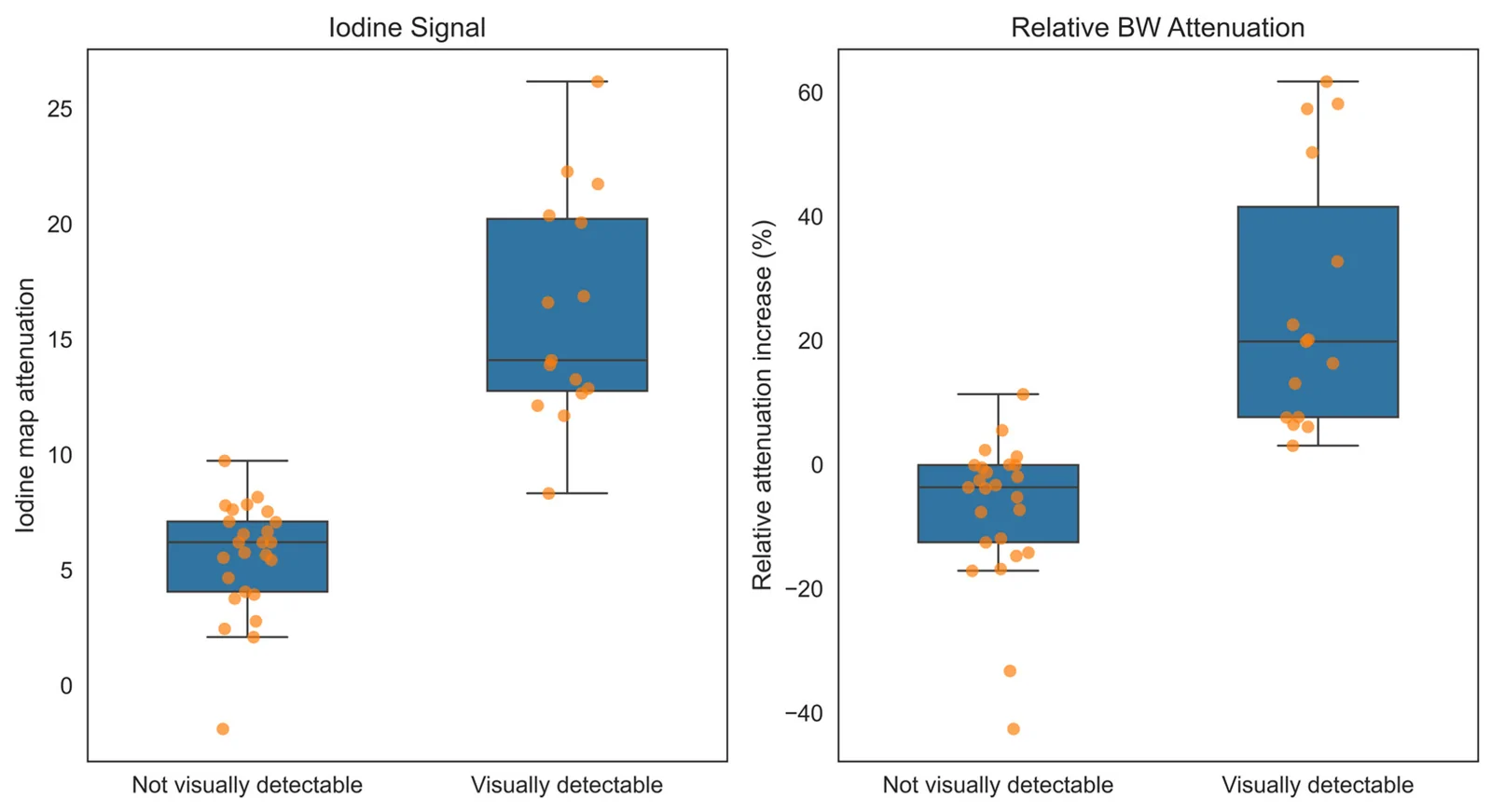
Distribution of IM attenuation values (**left**) and relative BW attenuation increase (**right**) according to visual detectability on standard BW images.

Within the 40 IM-positive infarct regions, 15 regions were visually detectable on BW images and 25 were visually undetectable. Exploratory analyses showed that quantitative attenuation values differed substantially between these groups. IM attenuation was higher in visually detectable regions than in regions visually undetectable on BW images, with a median attenuation of 14.10 HU (IQR, 12.77–20.22) versus 6.21 HU (IQR, 4.06–7.10), respectively (Mann–Whitney U = 374, *p* < 0.001) (Figure 2, left). Relative BW attenuation increase showed a similar difference, with a median of 19.83% (IQR, 7.64–41.53%) in visually detectable regions compared with −3.64% (IQR, −12.53 to −0.08%) in regions visually undetectable (Mann–Whitney U = 369, *p* < 0.001) (Figure 2, right).

In visually detectable regions, BW attenuation was significantly higher in the infarct ROIs than in the contralateral ROIs (median paired difference, 7.60 HU; *p* < 0.001). In regions visually undetectable on BW images, BW attenuation was significantly lower in infarct ROIs than in contralateral ROIs (median paired difference, −1.63 HU; *p* = 0.002). For IM attenuation, infarct-to-contralateral differences were significant in both visually detectable regions (mean paired difference, 14.45 HU; 95% CI, 11.87–17.02; *p* < 0.001) and regions visually undetectable on BW images (mean paired difference, 3.22 HU; 95% CI, 1.88–4.56; *p* < 0.001).

### 3.4. Determination of Perceptual Detectability Thresholds

**Figure 3:**
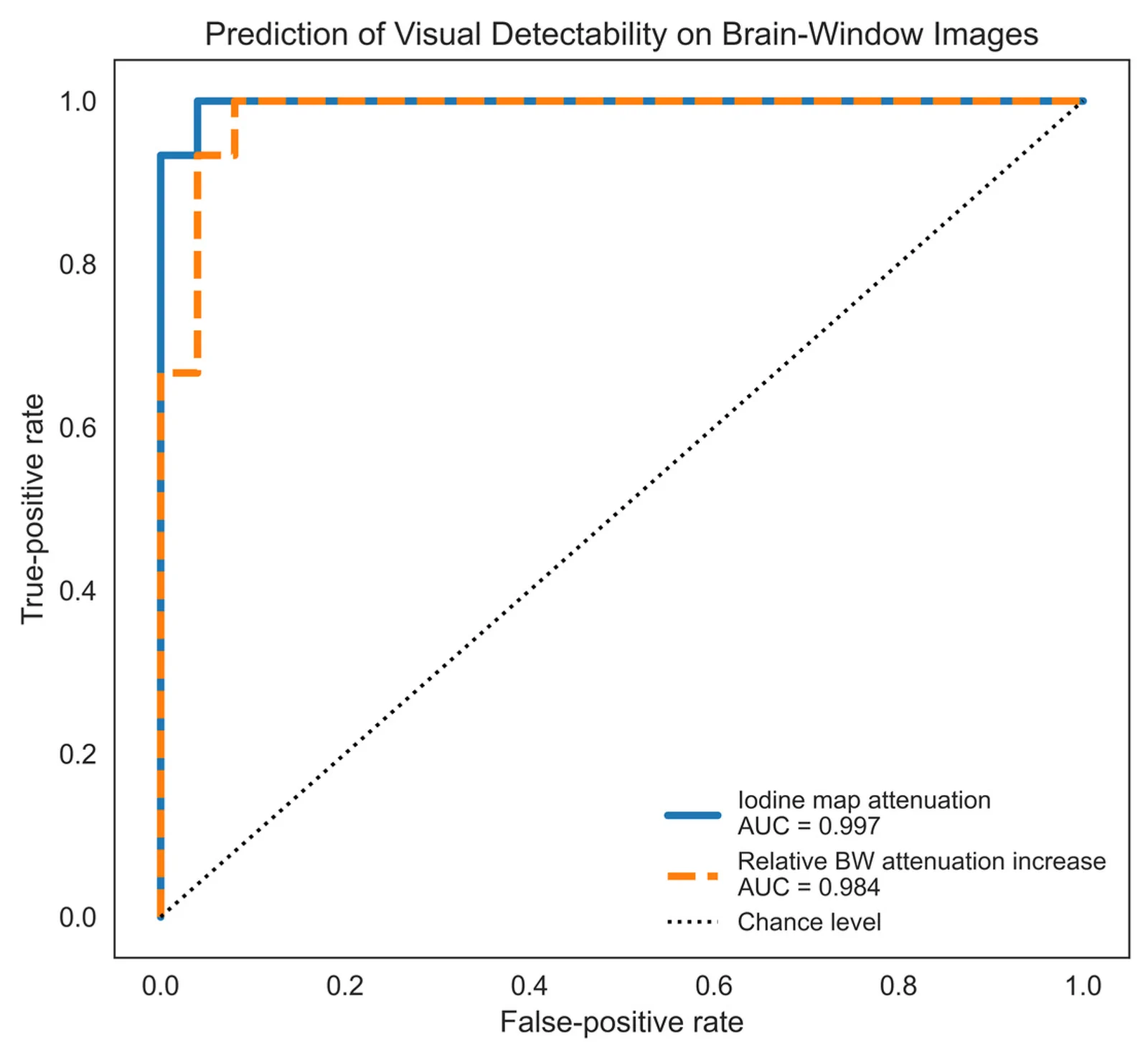
ROC curves for the prediction of visual detectability on BW images using IM attenuation and relative BW attenuation increase (%ΔHU).

**Figure 4:**
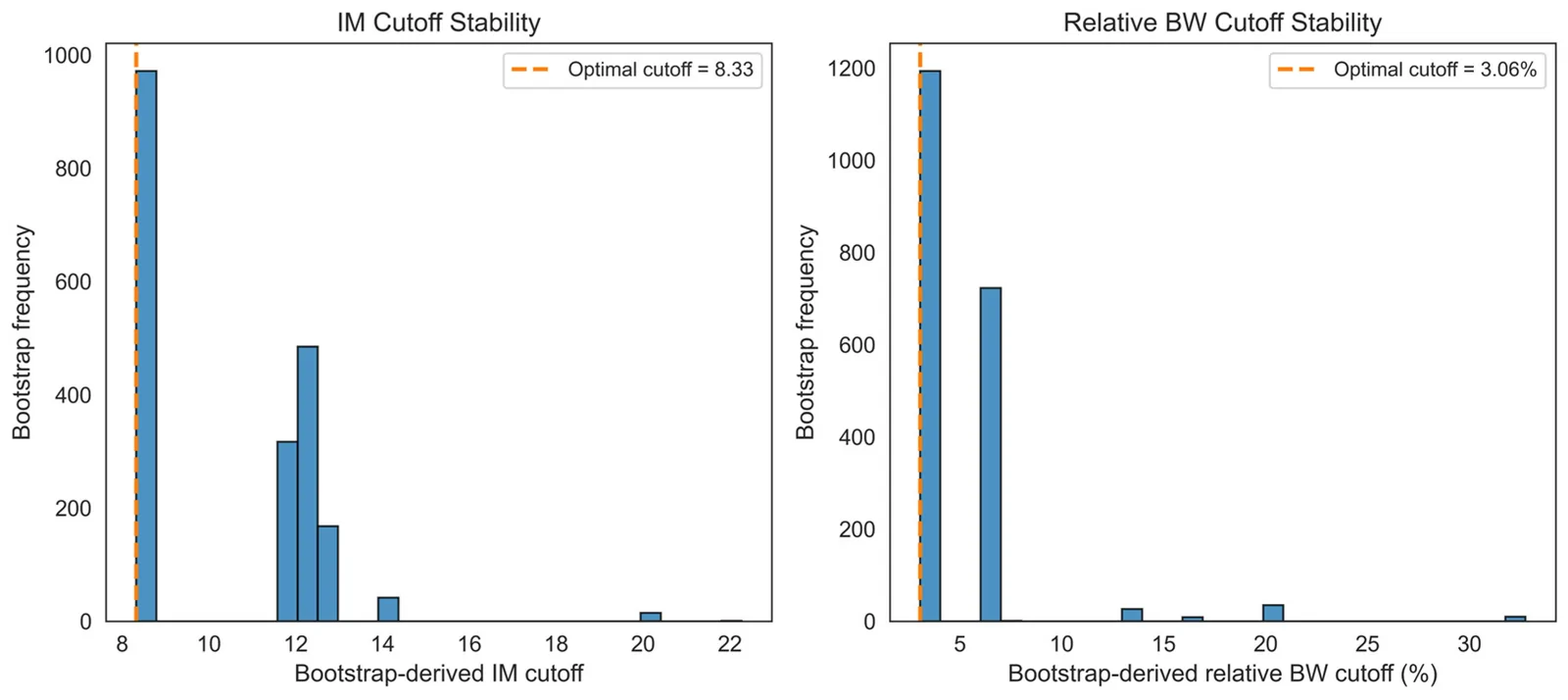
Cutoff stability assessed by patient-level bootstrap resampling. Distribution of Youden-index-derived cutoffs across 2000 bootstrap samples. The vertical line indicates the optimal cutoff in the original cohort.

ROC analysis using IM values to predict visual detectability on BW images within the 40 IM-positive ASPECTS regions yielded an AUC of 0.997 (Figure 3). Clustered bootstrap resampling at the patient level resulted in a 95% CI of 0.973–1.000. The optimal cutoff in the original cohort, determined by the Youden index, was 8.33 HU. At this threshold, sensitivity was 100%, specificity was 96.0%, PPV was 93.8%, and NPV was 100%. Across 2000 patient-level bootstrap samples, the median Youden-derived cutoff was 11.69 HU, with a 95% percentile interval of 8.33–12.67 HU, indicating variability in the internally derived threshold across bootstrap samples (Figure 4).

ROC analysis, using relative BW attenuation increase (%ΔHU), also demonstrated excellent discrimination, with an AUC of 0.984 (bootstrap 95% CI, 0.956–1.000) (Figure 3). The optimal cutoff in the original cohort was a relative attenuation increase of 3.06%. At this threshold, sensitivity was 100%, specificity was 92.0%, PPV was 88.2%, and NPV was 100%. Across bootstrap samples, the median Youden-derived cutoff was 3.06%, with a 95% percentile interval of 3.06–20.16%, demonstrating greater cutoff variability than the IM-based measure (Figure 4).

**Figure 5:**
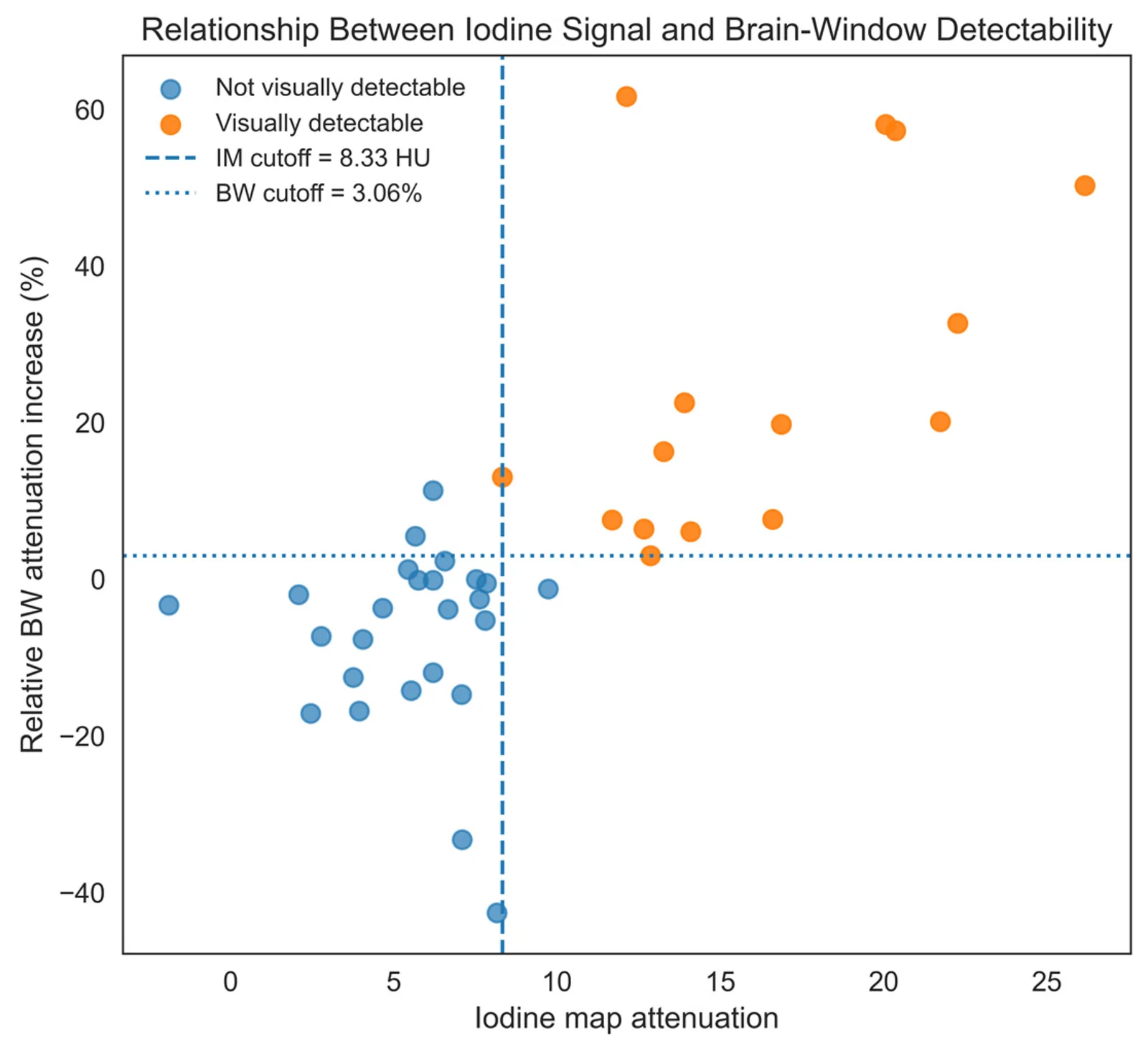
Relationship between IM attenuation and relative BW attenuation increase according to visual detectability of parenchymal hyperattenuation. Dashed lines indicate the optimal thresholds derived from ROC analysis.

Both quantitative parameters showed excellent discrimination of visually detectable versus visually undetectable BW hyperattenuation within IM-positive regions (Figure 5). The IM-derived threshold demonstrated a narrower bootstrap interval than the relative BW attenuation threshold, whereas both thresholds remained associated with uncertainty given the small number of independent patients.

## 4. Discussion

In this study, we investigated quantitative thresholds at which contrast agent accumulation becomes visually detectable on BW CT images, following MT. We found that infarct regions demonstrated a substantially increased iodine signal compared with anatomically matched contralateral brain tissue, whereas absolute attenuation differences on standard BW images were not significantly different at the group level. This highlights the limited sensitivity of conventional grayscale assessment for detecting early post-interventional parenchymal alterations. While prior studies have primarily focused on iodine-related attenuation thresholds for hemorrhage prediction, our findings extend this framework by linking quantitative iodine concentration directly to human visual perception on conventional CT images. Among the 40 IM-positive ASPECTS regions demonstrating hyperattenuation on IM, 15 were visually detectable on BW images, whereas 25 were visually undetectable. Quantitative IM attenuation and relative BW attenuation increase both showed excellent discrimination between visually detectable and visually undetectable regions, with AUCs of 0.997 and 0.984, respectively. The corresponding Youden-derived thresholds were 8.33 HU for IM attenuation and 3.06% for relative BW attenuation increase. The patient-level bootstrap analysis yielded 95% confidence intervals of 0.973–1.000 for the IM AUC and 0.956–1.000 for the relative BW AUC. The bootstrap intervals for the corresponding cutoffs were 8.33–12.67 HU and 3.06–20.16%, respectively, indicating greater stability of the IM-derived threshold.

The greater stability of IM-derived thresholds compared with relative BW attenuation may be explained by the fundamentally different information represented by the two measures. Material decomposition on IM is designed to isolate the iodine-related component of attenuation, thereby providing a more direct quantitative measure of contrast accumulation. In contrast, attenuation on conventional BW images reflects the composite signal of brain tissue and iodine and is additionally influenced by image noise and local variations in baseline parenchymal attenuation. Moreover, BW attenuation increase (%ΔHU) is derived from the relationship between measurements in the affected and contralateral hemispheres, introducing variability from both measurements into the resulting metric. These factors may contribute to the wider bootstrap interval observed for the BW-derived threshold compared with the IM-derived threshold in our study. Direct iodine quantification may therefore provide a more robust and reproducible measure of post-interventional contrast accumulation than attenuation differences derived from conventional BW images. Similar quantitative approaches have been reported in DECT studies, where iodine concentration thresholds were shown to stratify the risk of intracranial hemorrhage after MT, further supporting the clinical relevance of iodine-based cutoffs [12]. The relative attenuation increase on BW images also demonstrated strong predictive performance. However, threshold variability was substantially greater compared with iodine-based measurements, suggesting that iodine quantification provides a more stable surrogate of perceptual visibility. Together, these findings provide preliminary evidence for a potential link between spectral iodine measurements and human visual perception on conventional CT imaging. Early post-MT hyperattenuation reflects a complex interplay of contrast staining, BBB disruption, and evolving ischemic injury [3,13,14]. While iodine accumulation may be readily detectable on spectral reconstructions, its visibility on routine BW images depends on whether attenuation differences exceed the perceptual limits of grayscale discrimination. This concept is consistent with prior DECT studies showing that quantitative iodine extravasation correlates with post-interventional complications, where higher iodine concentrations were associated with an increased risk of hemorrhagic transformation [5].

The visibility-stratified analysis further supports this interpretation. Regions that were visually detectable on BW images showed substantially higher IM attenuation than regions that were visually undetectable (median, 14.10 vs. 6.21 HU; *p* < 0.001). Similarly, the relative BW attenuation increase was markedly higher in visually detectable regions (median, 19.83% vs. −3.64%; *p* < 0.001). Within visually detectable regions, BW attenuation was significantly higher than in the corresponding contralateral regions (median paired difference, 7.60 HU; *p* < 0.001), whereas BW attenuation was significantly lower than the contralateral reference in regions that were visually undetectable (median paired difference, −1.63 HU; *p* = 0.002). In contrast, IM attenuation was significantly higher than the contralateral reference in both groups, with mean paired differences of 14.45 HU and 3.22 HU in visually detectable and visually undetectable regions, respectively. These findings indicate that iodine accumulation may be quantitatively detectable on IM even when it does not result in visually appreciable hyperattenuation on conventional BW images.

Our results demonstrate that visually detectable hyperattenuation on unenhanced CT is not primarily determined by absolute brain attenuation values, but rather by sufficiently elevated iodine concentration within affected tissue. Importantly, infarct regions exhibited a markedly increased iodine signal even when corresponding BW images appeared visually inconspicuous. This finding explains a frequently encountered clinical scenario in which IM reveals marked hyperattenuation that remains subtle or invisible on routine reconstructions. The strong separation between visually detectable and visually undetectable regions according to IM attenuation further suggests that the amount of iodine accumulation is an important determinant of whether post-interventional parenchymal hyperattenuation becomes apparent on conventional BW images.

A quantitative iodine threshold associated with visual detectability could support standardized interpretation across readers and institutions by providing an objective reference for expected visibility on conventional images. In this pilot cohort, IM values above the internally derived cutoff of 8.33 HU were associated with a higher likelihood of visual detectability on routine BW reconstructions, whereas lower iodine concentrations may remain visually undetectable despite measurable contrast accumulation. The relatively wide bootstrap interval for the BW-derived threshold (3.06–20.16%) compared with that of the IM-derived threshold (8.33–12.67 HU) suggests that the latter may provide a more consistent quantitative descriptor of perceptual visibility in this cohort. Moreover, the observed high discriminatory performance and greater threshold stability of iodine-based thresholds suggest that PCCT-derived iodine imaging may reduce reliance on subjective visual assessment and improve reproducibility in post-MT imaging evaluation.

Previous studies using DECT and spectral CT techniques have demonstrated that iodine mapping enables differentiation between hemorrhage and contrast staining and may predict infarct evolution after endovascular therapy [5,6]. Moreover, DECT has been shown to substantially alter the assessment of post-MT hemorrhagic complications, affecting clinical decision-making in up to one-third of cases [15], underscoring the practical importance of spectral imaging in acute stroke workflows. However, prior work has primarily focused on tissue outcome prediction or material characterization rather than perceptual detectability [3,13,14,16]. To the best of our knowledge, the present study is among the first to quantitatively define the relationship between iodine concentration and human visual detectability on conventional BW images. By linking spectral quantification with observer perception, our findings extend prior research toward potentially clinically actionable interpretation thresholds.

Several limitations should be acknowledged. The retrospective design may have introduced selection bias. However, according to our institutional protocol, all patients undergoing MT routinely receive post-interventional PCCT. Thus, the use of PCCT was not based on patient characteristics, procedural factors, or clinical status. Inclusion in the present study was restricted to patients with available post-interventional PCCT and FU imaging. The sample size was therefore determined by the number of eligible patients during the study period. The resulting small sample size did not permit meaningful sex-specific analyses. Therefore, potential sex-related differences in iodine staining, visual detectability, or attenuation thresholds could not be evaluated.

Importantly, the perceptual threshold analysis was restricted to the 40 ASPECTS regions showing hyperattenuation on IM. Therefore, the reported AUCs and derived cutoffs characterize BW detectability conditional on the presence of an IM abnormality and should not be interpreted as thresholds for detecting infarction across all post-MT ASPECTS regions. IM-negative regions that subsequently evolved into infarction were not included in the perceptual threshold analysis. Analyses were performed at the ASPECTS-region level, with the three replicate ROI measurements averaged to obtain a single representative value for each region and paired with the corresponding contralateral reference region. Although patient-level clustered bootstrap resampling was used to account for within-patient correlation and to estimate uncertainty, the 40 analyzed regions originated from only 12 patients, and only 15 regions were visually detectable on BW images. Bootstrap resampling provides an estimate of statistical uncertainty but does not constitute independent validation. The reported AUCs and Youden-derived thresholds of 8.33 HU for IM attenuation and 3.06% for relative BW attenuation increase should therefore be regarded as internally derived pilot estimates rather than validated diagnostic or universal perceptual thresholds. The relatively wide bootstrap interval for the BW-derived threshold further emphasizes its uncertainty. External validation in larger, independent multicenter cohorts is required before these values can be considered for broader clinical application.

Detectability assessment was based on visual classification by experienced readers under standardized viewing conditions. Visual perception may vary depending on display settings, windowing parameters, and reader experience, which may influence the practical applicability of the derived thresholds. Nevertheless, inter-reader agreement was excellent for both BW visibility (κ = 0.86) and IM assessment (κ = 0.81), supporting the reproducibility of the visual classification in the present cohort. Further, the thresholds identified in this study represent internally derived perceptual cutoffs rather than universally applicable biological constants. Generalizability may also be limited because all examinations were performed on a single photon-counting CT platform (NAEOTOM Alpha Peak, Siemens Healthineers). Future multicenter studies are warranted to evaluate the robustness of these thresholds across different scanners, reconstruction settings, and clinical environments.

## 5. Conclusions

Among post-MT ASPECTS regions showing hyperattenuation on IM, PCCT-derived iodine quantification enables objective assessment of visual detectability on conventional BW images. In this cohort, IM attenuation of 8.33 HU and a relative BW attenuation increase of 3.06% were associated with visual detectability of parenchymal hyperattenuation, with iodine-based measurements demonstrating greater threshold stability across patient-level bootstrap resampling. These findings provide preliminary evidence for a potential link between spectral iodine measurements and human visual perception and may support more standardized interpretation of early post-MT brain CT imaging. The identified thresholds should be considered internally derived candidate thresholds and require validation in larger, independent multicenter cohorts, with potential scanner-specific variability taken into account.

## Data Availability

The data presented in this study are available on request from the corresponding author due to ethical reasons.

## Supplementary Materials

The following supporting information can be downloaded at https://www.mdpi.com/article/10.3390/diagnostics16182915/s1, Table S1: Patient, stroke, and therapy characteristics.

## Abbreviations

The following abbreviations are used in this manuscript:

ASPECTS	Alberta Stroke Program Early CT Score
AUC	Area Under the Receiver Operating Characteristic Curve
BBB	Blood–Brain Barrier
BW	Brain Window
CI	Confidence Interval
CT	Computed Tomography
DECT	Dual-Energy Computed Tomography
EID	Energy-Integrating Detector
FU	Follow-Up
HU	Hounsfield Unit(s)
IM	Iodine Map
IQR	Interquartile Range
LVO	Large Vessel Occlusion
MT	Mechanical Thrombectomy
NCCT	Non-Contrast Computed Tomography
NPV	Negative Predictive Value
PCCT	Photon-Counting Computed Tomography
PPV	Positive Predictive Value
ROC	Receiver Operating Characteristic
ROI	Region of Interest
SD	Standard Deviation
VNC	Virtual Non-Contrast
%ΔHU	Relative Percentage Increase in Hounsfield Units (relative attenuation increase)
κ	Cohen’s Kappa Coefficient
