# Supplementary material for: From Iodine Maps to Brain Window Images: Quantitative Visibility Thresholds of Iodine Staining Using Photon-Counting CT—A Retrospective Observational Study
Source: Diagnostics (Basel). 2026 Sep 9;16(18):2915. doi: 10.3390/diagnostics16182915 (PMC13605841; doi:10.3390/diagnostics16182915)
Supplement: Supplementary file 1 [file diagnostics-16-02915-s001.zip › diagnostics-4487180-supplementary.pdf]

**Supplementary Table S1** Patient, stroke, and therapy characteristics.

| Pt No | Sex | Age (y) | Onset to MT (min) | NIHSS | i.v. thrombolysis | Stroke etiology | Occlusion location | Initial ASPECTS | Final ASPECTS | Initial eTICI | Final eTICI | MT method | Heidelberg bleeding classification |
|---|---|---|---|---|---|---|---|---|---|---|---|---|---|
| 1 | m | 63 | 190 | 22 | yes | cryptogenic | M1 distal | 9 | 8 | 0 | 2b65 | A&SR | no bleeding |
| 2 | f | 75 | 225 | 22 | yes | atrial fibrillation | Carotid T (L) | 10 | 7 | 0 | 3 | SR | no bleeding |
| 3 | f | 81 | 310 | 15 | no | cryptogenic | Carotid T (T) | 10 | 0 | 0 | 2c | SR | no bleeding |
| 4 | m | 47 | 155 | 21 | yes | cryptogenic | Carotid T (L) | 10 | 5 | 0 | 2b65 | A&SR | 3c |
| 5 | f | 66 | 226 | 18 | yes | atrial fibrillation | M1 mid | 10 | 9 | 0 | 3 | A&SR | no bleeding |
| 6 | f | 87 | 279 | 6 | yes | ICA stenosis | M1 distal | 9 | 7 | 0 | 2b65 | SR | 3c |
| 7 | f | 78 | 196 | 18 | yes | aortic plaque | Carotid T (T) | 10 | 8 | 0 | 3 | A&SR | no bleeding |
| 8 | m | 87 | 577 | 11 | no | cryptogenic | M1 proximal | 9 | 8 | 0 | 3 | A&SR | HI1 |
| 9 | f | 73 | 240 | 20 | yes | atrial fibrillation | M2 | 10 | 8 | 0 | 2b65 | SR | no bleeding |
| 10 | m | 58 | 540 | 13 | no | cryptogenic | M1 distal | 4 | 4 | 0 | 3 | A&SR | no bleeding |
| 11 | m | 82 | 250 | 18 | yes | cryptogenic | M1 proximal | 10 | 0 | 0 | 2b65 | A&SR | no bleeding |
| 12 | m | 57 | 225 | 15 | no | ICA stenosis | M1 proximal | 8 | 4 | 0 | 2b65 | A&SR | no bleeding |

Abbreviations: Pt No, patient number; m, male; f, female; y, years; MT, mechanical thrombectomy; min, minutes; i.v., intravenous; ASPECTS, Alberta Stroke Program Early CT Score; eTICI, expanded Thrombolysis in Cerebral Infarction; M1, first segment of the middle cerebral artery; M2, second segment of the middle cerebral artery; ICA, internal carotid artery; Carotid-T, carotid T occlusion (terminal ICA occlusion involving the

origins of the MCA and ACA); Carotid-L, carotid L occlusion (terminal ICA occlusion involving the MCA origin, with preservation of the ACA origin); SR, stent retriever; A, aspiration.
